# Comparative performance evaluation of large language models in answering esophageal cancer-related questions: a multi-model assessment study

**DOI:** 10.3389/fdgth.2025.1670510

**Published:** 2025-10-07

**Authors:** Zijie He, Lilan Zhao, Genglin Li, Jintao Wang, Songyu Cai, Pengjie Tu, Jingbo Chen, Jianman Wu, Juan Zhang, Ruiqi Chen, Yangyun Huang, Xiaojie Pan, Wenshu Chen

**Affiliations:** ^1^ Department of Thoracic Surgery, Shengli Clinical Medical College of Fujian Medical University, Fujian Provincial Hospital, Fuzhou University Affiliated Provincial Hospital, Fuzhou, China; ^2^ Department of Cardiothoracic Surgery, Tianjin Medical University General Hospital, Tianjin, China; ^3^ Department of Medical Oncology, Shengli Clinical Medical College of Fujian Medical University, Fujian Provincial Hospital, Fuzhou University Affiliated Provincial Hospital, Fuzhou, China; ^4^ Department of Radiology, Shengli Clinical Medical College of Fujian Medical University, Fujian Provincial Hospital, Fuzhou University Affiliated Provincial Hospital, Fuzhou, China

**Keywords:** large language models, artificial intelligence, esophageal cancer, medical question answering, medical education

## Abstract

**Background:**

Esophageal cancer has high incidence and mortality rates, leading to increased public demand for accurate information. However, the reliability of online medical information is often questionable. This study systematically compared the accuracy, completeness, and comprehensibility of mainstream large language models (LLMs) in answering esophageal cancer-related questions.

**Methods:**

In total, 65 questions covering fundamental knowledge, preoperative preparation, surgical treatment, and postoperative management were selected. Each model, namely, ChatGPT 5, Claude Sonnet 4.0, DeepSeek-R1, Gemini 2.5 Pro, and Grok-4, was queried independently using standardized prompts. Five senior clinical experts, including three thoracic surgeons, one radiologist, and one medical oncologist, evaluated the responses using a five-point Likert scale. A retesting mechanism was applied for the low-scoring responses, and intraclass correlation coefficients were used to assess the rating consistency. The statistical analyses were conducted using the Friedman test, the Wilcoxon signed-rank test, and the Bonferroni correction.

**Results:**

All the models performed well, with average scores exceeding 4.0. However, the following significant differences emerged: Gemini excelled in accuracy, while ChatGPT led in completeness, particularly in surgical and postoperative contexts. Minor differences appeared in fundamental knowledge, but notable disparities were found in complex areas. Retesting showed improvements in overall quality, yet some responses showed decreased completeness and relevance.

**Conclusion:**

Large language models have considerable potential in answering questions about esophageal cancer, with significant differences in completeness. ChatGPT is more comprehensive in complex scenarios, while Gemini excels in accuracy. This study offers guidance for selecting artificial intelligence tools in clinical settings, advocating for a tiered application strategy tailored to specific scenarios and highlighting the importance of user education to understand the limitations and applicability of LLMs.

## 1 Introduction

Esophageal cancer (EC), involving malignant tumors, is characterized by significant regional disparities and represents a considerable burden globally. According to GLOBOCAN 2020, it is the seventh most prevalent malignancy and the sixth leading cause of cancer-related deaths worldwide (1). The disease is geographically concentrated, with high incidence rates primarily in China, East Asia, Central Asia, the Middle East, and parts of East Africa. Notably, China accounts for over 50% of global cases (2). As the disease burden intensifies, public demand for reliable medical information on esophageal cancer grows. However, traditional sources of medical information are often inadequate, with online content varying in quality and lacking guaranteed medical accuracy, potentially misleading patients and adversely affecting clinical practice.

In recent years, the rapid development of large language models (LLMs) has opened new avenues for acquiring medical information. Preliminary studies have validated the application value of LLMs in medical question-and-answer settings. For instance, Riestra-Ayora et al. evaluated the performance of ChatGPT in addressing common ear, nose, and throat conditions, finding that it provides clear and concise information along with treatment suggestions (3). In addition, other research has explored the potential of ChatGPT 4.0 in clinical decision support for esophageal diseases (4). However, these studies primarily focused on evaluating a single model and lacked systematic comparative analyses of multiple models. Furthermore, significant performance differences may arise from disparities in training data sources and algorithmic architecture designs.

Therefore, this study aims to systematically evaluate the performance of five mainstream LLMs in answering common questions from patients with esophageal cancer. By employing a multidimensional assessment framework, the research will analyze the strengths and limitations of each model in different clinical scenarios, helping to identify reliable sources of information for patients seeking knowledge about esophageal cancer and providing a scientific basis for selecting and applying artificial intelligence (AI) tools in clinical practice.

## 2 Methods

### 2.1 Data collection

This study systematically collected common questions from patients with esophageal cancer. The sources of these questions included the official websites of major medical institutions, social media platforms, online health consultation platforms, and typical questions of concern found in related academic literature. Detailed information on the sources is provided in Supplementary Table S1. During the screening process, we excluded certain questions based on the following criteria:

1. Duplicate or similarly worded questions, with only the most representative retained for analysis.
2. Questions involving significant individual variations precluding standardized responses (e.g., “What is my cancer stage?”).
3. Patient concerns unrelated to the disease (e.g., “If I am worried about treatment costs, who can help me?”).

After filtering, we standardized the wording of some questions to ensure clarity and consistency. The final collected questions were categorized into four main groups based on the following clinical diagnostic and treatment processes and patient concerns: basic knowledge of esophageal cancer, preoperative preparation, surgical treatment, and postoperative rehabilitation management. Since this study did not involve patient recruitment, approval from the institutional ethics committee was not required.

### 2.2 Large language model overview

#### 2.2.1 ChatGPT 5

ChatGPT is an LLM developed by OpenAI based on the generative pre-trained transformer (GPT) architecture. Compared to its predecessors, ChatGPT 4.0 demonstrates significant advancements in model scale, training data quality, multimodal processing, and reasoning capabilities. The model can acquire data from a wide range of online resources and continuously optimize response quality through reinforcement learning with human feedback (RLHF). It can incorporate human input during conversations and adjust responses based on feedback, while engaging in ongoing learning to mitigate potential biases in generated answers (5). ChatGPT has diverse applications in the medical field, including assisting in medical report writing, answering exam questions, and aiding in disease diagnosis and patient consultations, particularly excelling in health information validation and providing personalized medical advice (6–9). The latest version, ChatGPT 5.0, further enhances its code generation, complex reasoning, and long-context processing capabilities, with an emphasis on its potential in medical research and clinical decision support during its initial rollout.

#### 2.2.2 Claude Sonnet 4.0

Claude is an LLM developed by Anthropic, with its Claude 3.5 Sonnet version demonstrating outstanding performance in natural language understanding, safety, and accuracy. Compared to other LLMs, Claude 3.5 Sonnet has unique advantages in handling complex reasoning tasks and providing balanced, nuanced responses, making it particularly suitable for medical question-and-answer scenarios that require high accuracy and ethical considerations. This model emphasizes constitutional AI training methods, enhancing its reliability in sensitive areas such as clinical decision support and demonstrating impressive performance in evaluations when compared to medical professionals (10–13). The latest version, Claude Sonnet 4.0, features improvements in following complex instructions, with clear reasoning and mixed reasoning capabilities, and extended context windows, further increasing its potential for use in medical diagnostics, patient education, and clinical benchmark assessments.

#### 2.2.3 DeepSeek-R1

DeepSeek is a series of open-source LLMs, including the primary versions DeepSeek-V3 and DeepSeek-R1, which play a significant role in the field of LLMs and offer new opportunities for applications in specialized areas such as healthcare. These models contain approximately 500 billion parameters, making them among the largest language models currently available. Unlike proprietary models, such as the GPT series models, a key advantage of DeepSeek models is their open-source nature, providing transparency and the ability to operate within institutional IT environments at a significantly lower cost. Notably, DeepSeek-R1 is equipped with explicit reasoning capabilities, enhancing its reasoning skills through reinforcement learning. This ability makes it particularly suited for tasks requiring complex logical reasoning, demonstrating potential value in clinical decision-making and medical benchmark testing (14–16).

#### 2.2.4 Gemini 2.5 Pro

Gemini is a multimodal large language model developed by Google DeepMind. Early versions, such as Gemini Advanced and Gemini, have shown considerable value in medical applications. For example, studies have conducted clinical pathological analyses in gastrointestinal pathology, compared Gemini’s performance with ChatGPT in ophthalmology licensing examinations, and performed multidimensional assessments in dental implant clinical settings (17, 18). These models excel at addressing complex medical inquiries, providing differential diagnoses, and interpreting pathological results. The latest version, Gemini 2.5 Pro, further advances scientific understanding and enhances long-context processing capabilities, enabling deeper research and improved visualization of health indicators. It is anticipated to demonstrate superior performance in medical testing.

#### 2.2.5 Grok-4

Grok is a reasoning-oriented large language model developed by xAI, with Grok-3 showing promise in medical decision assessment and being acknowledged for its ability to surpass human experts (19, 20). The updated version, Grok 4, has reached new heights in benchmark testing, achieving higher scores on abstraction and reasoning corpus for artificial general intelligence ARC-AGI-2 and artificial intelligence in medicine AIME 2025. In addition, it further optimizes high-parameter training designs, increasing the scope of its application in healthcare, science, and clinical decision support.

### 2.3 Response generation

This study rigorously followed the principles of blinding and utilized a collaborative division of labor within the research team. GL was responsible for the systematic collection and scientific screening of clinical questions, while ZH managed the standardized execution of model queries. The submission of questions began in August 2025. To ensure the professionalism and consistency of model outputs, the research team established a standardized role-setting protocol. Prior to each model query, the uniform instruction “Please answer my question as an expert in esophageal cancer medicine” was provided to elicit the model's specialized knowledge and enhance the clinical relevance of its responses.

To guarantee the independence and reproducibility of data collection, a strict dialog isolation strategy was implemented; each clinical question was treated as an independent dialog instance and submitted using the “New Conversation” function in each model platform. This approach ensured there was no information transfer or interference between questions, mitigating any potential impact of dialog history on the quality of the models’ responses. All the models’ answers were recorded in a standardized format.

### 2.4 Expert scoring and feedback

To ensure the objectivity and professionalism of the evaluation results, the study formed a scoring panel consisting of five senior clinical experts, including three thoracic surgeons, one radiologist, and one medical oncologist. Each scoring expert had at least 10 years of relevant clinical experience and possessed extensive practical experience in the diagnosis, treatment, or management of esophageal cancer. The scoring process utilized a standardized five-point Likert scale to independently assess the responses of each model. To avoid potential bias, the experts were not informed of the specific sources of each response during the evaluation. The scoring dimensions were divided into the following three aspects:

1. Accuracy: Medical accuracy of the provided information (1 = completely incorrect, 2 = more incorrect than correct, 3 = equally correct and incorrect, 4 = more correct than incorrect, 5 = completely correct).
2. Completeness: Completeness of the provided information (1 = very incomplete, 2 = incomplete, 3 = basically complete, 4 = relatively complete, 5 = very complete).
3. Comprehensibility: Comprehensibility of the provided information (1 = very difficult to understand, 2 = difficult to understand, 3 = partially understandable, 4 = relatively easy to understand, 5 = very easy to understand).

**Figure 1:**
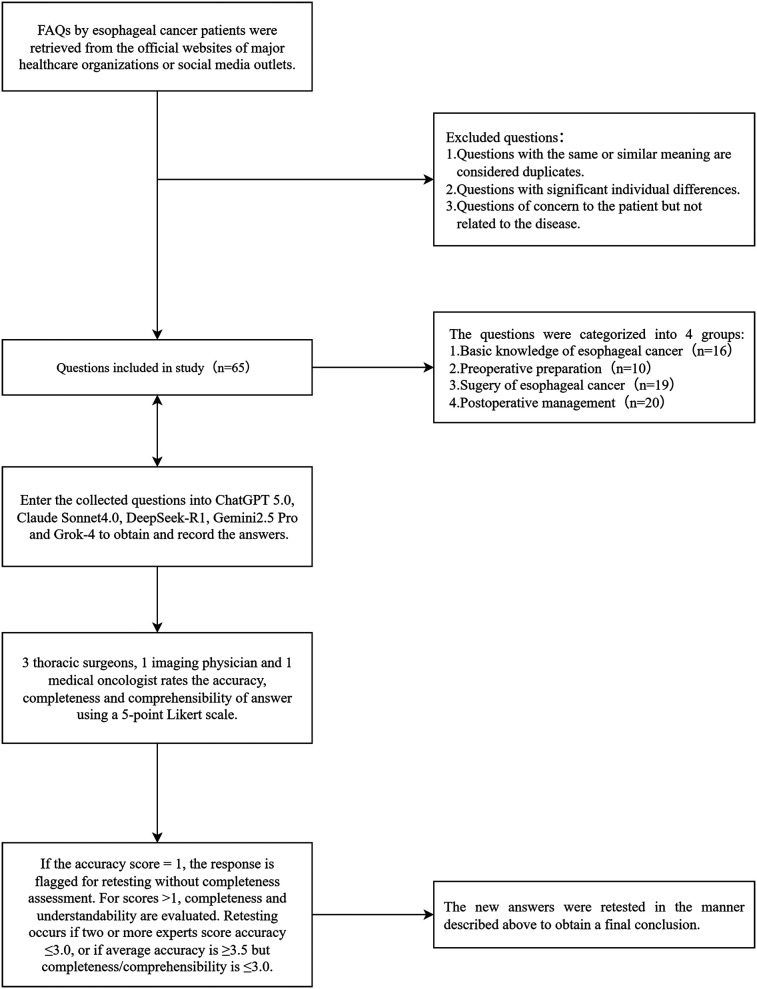
Flowchart of the study, including the selection process for questions related to esophageal cancer. These questions were sourced from the official websites of major hospitals and reputable institutions. FAQs, frequently asked questions.

In terms of quality control measures, this study introduced a retesting mechanism. Specifically, the experts first scored the responses for accuracy; if the accuracy score was 1 point, the completeness assessment was not conducted, and the response was directly flagged for retesting. For responses with accuracy scores >1, the evaluation continued with the completeness and comprehensibility assessments. Subsequently, based on the scores from all the experts, the following retesting criteria were applied: if two or more experts rated the accuracy of the same question at 3 points or lower, or if the average accuracy score for a question was ≥3.5 but the average completeness or comprehensibility score was ≤3.0, a retest was required (21). Retesting involved re-querying the corresponding model after 1 week, with the experts re-evaluating the response using identical criteria. Questions with persistently unsatisfactory scores were flagged and analyzed in detail. The research workflow is shown in Figure 1.

### 2.5 Statistical analysis

The statistical analysis was performed using SPSS version 27.0. Descriptive statistics are reported as mean ± standard deviation (SD) for dimension scores. Inter-rater reliability was assessed using the intraclass correlation coefficient (ICC), with reliability interpreted as follows: ICC < 0.5 (poor), 0.5–0.74 (moderate), 0.75–0.9 (good), and >0.9 (excellent). For the comparative analysis of the five LLMs, the Friedman test was employed to evaluate overall differences across the assessment dimensions, with a significance level set at *P* < 0.05. When the Friedman test yielded significant results, the Wilcoxon signed-rank test was applied for pairwise comparisons, and the Bonferroni correction was implemented to control for Type I errors due to multiple comparisons. For questions that required retesting, the Wilcoxon rank-sum test was used to compare score differences before and after retesting. All the statistical tests were two-tailed, and a *P*-value of <0.05 was considered statistically significant.

## 3 Results

### 3.1 Question collection and classification

**Figure 2:**
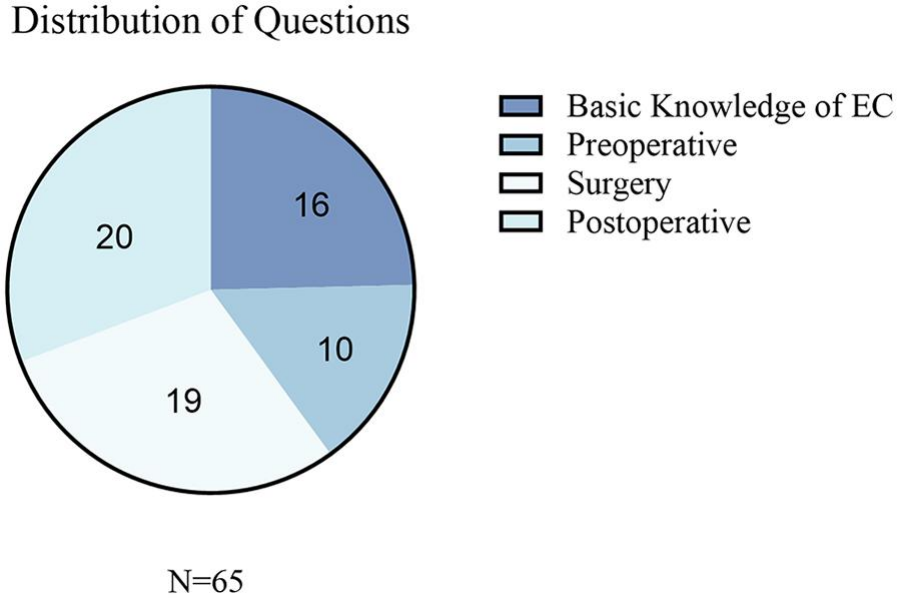
Distribution of the collected questions related to EC. This figure presents the categorization of the questions collected in the study. EC, esophageal cancer.

A total of 65 esophageal cancer-related clinical questions were included in the evaluation and were classified into the following four core esophageal cancer clinical management domains: (1) knowledge (etiology, epidemiology, and pathological classification), (2) preoperative preparation (diagnostic assessment, staging examination, and perioperative preparation), (3) surgical management (surgical indications, approach selection, and complication management), and (4) postoperative management (rehabilitation guidance, follow-up monitoring, and recurrence prevention). The distribution and proportion of the questions across the domains are shown in Figure 2. All 65 questions and the corresponding detailed responses from the five LLMs are provided in Supplementary Table S2.

### 3.2 Retest results and model performance changes

**Figure 3:**
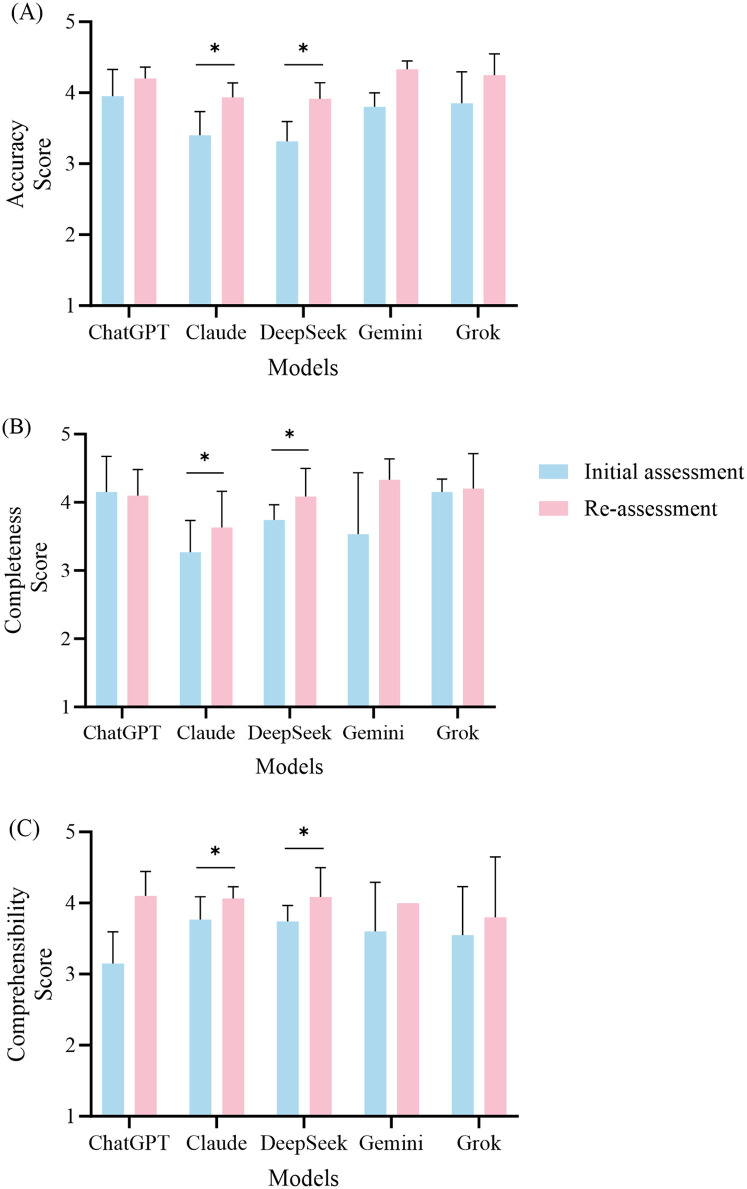
Performance comparison of the five large language models in the initial and re-assessment phases. This figure illustrates the mean scores (± standard deviation) for accuracy (**A**), completeness (**B**), and comprehensibility (**C**) across the five models, namely, ChatGPT, Claude, DeepSeek, Gemini, and Grok. The blue bars indicate the scores from the initial assessment, while the red bars represent the scores from the re-assessment. Statistical analyses were performed using the Wilcoxon signed-rank test to compare the scores between the initial assessment and the re-assessment, with significant improvements indicated by asterisks.

According to the predetermined retesting criteria, retests were conducted for ChatGPT, Claude, DeepSeek, Gemini, and Grok, with four, six, seven, three, and four retested questions, respectively. All the retests were scored using the same evaluation criteria. The comparison of scores before and after retesting is shown in Figure 3, and the retested questions along with their answers can be found in Supplementary Table S3.

#### 3.2.1 ChatGPT retest performance

After the retests were conducted for ChatGPT, the accuracy score improved from 3.95 ± 0.38 to 4.20 ± 0.16 (*P* = 0.102). Although this difference did not reach statistical significance, it indicates a noticeable trend toward improvement. Regarding completeness, the average score for the initial tests was 4.15 ± 0.53, while the retest average score was 4.10 ± 0.38 (*P* = 0.785), showing no significant change in this dimension. The comprehensibility score improved from 3.15 ± 0.44 to 4.10 ± 0.35 (*P* = 0.066); however, this change was not statistically significant.

#### 3.2.2 Claude retest performance

The retest results for Claude indicated significant improvements across all assessment dimensions. The accuracy score increased from 3.40 ± 0.34 to 3.93 ± 0.21 (*P* = 0.027). In terms of completeness, the retest score was 3.63 ± 0.53, showing a significant improvement compared to the initial score of 3.27 ± 0.47 (*P* = 0.026). The comprehensibility score also increased significantly, increasing from 3.77 ± 0.32 to 4.07 ± 0.16 (*P* = 0.041).

#### 3.2.3 DeepSeek retest performance

DeepSeek also demonstrated significant improvements in the retest results. The accuracy score increased from 3.31 ± 0.28 to 3.91 ± 0.23 (*P* = 0.017). The completeness score increased from 3.74 ± 0.22 to 4.09 ± 0.41 (*P* = 0.026), while the comprehensibility score saw a notable increase from 3.51 ± 0.53 to 4.17 ± 0.53 (*P* = 0.016).

#### 3.2.4 Gemini retest performance

Gemini's retest results showed some improvement, with the accuracy score increasing from 3.80 ± 0.20 to 4.33 ± 0.11 (*P* = 0.102). In the completeness dimension, the initial score was 3.53 ± 0.90, while the retest score was 4.33 ± 0.31 (*P* = 0.180). The comprehensibility score increased from 3.60 ± 0.69 to 4.00 (*P* = 0.317). Although there were increases in the scores across all dimensions, they did not reach statistical significance.

#### 3.2.5 Grok retest performance

In the retest for Grok, the accuracy score increased from 3.85 ± 0.44 to 4.25 ± 0.30 (*P* = 0.066), indicating a trend toward statistical significance. The completeness score showed a slight improvement, rising from 4.15 ± 0.19 to 4.20 ± 0.52 (*P* = 0.705). The comprehensibility score increased from 3.55 ± 0.68 to 3.80 ± 0.85, but this change did not reach statistical significance (*P* = 0.461).

#### 3.2.6 Overall retest findings

In summary, the overall performance of the five models improved after the retesting, with Claude and DeepSeek notable in this regard. The retest results indicated that both Claude and DeepSeek demonstrated significant improvements across all the evaluation dimensions, while Grok exhibited a notable increase in accuracy. In contrast, the performance of ChatGPT and Gemini did not reach statistical significance after the retests.

### 3.3 Inter-rater reliability analysis

To ensure the reliability and scientific validity of the evaluation results, this study quantified the consistency levels among five evaluators for scoring the medical question-answering abilities of five large language models using ICC. The assessment framework comprises three dimensions, namely, accuracy, completeness, and comprehensibility, and the evaluation results are presented in Table 1.

**Table 1:** Intraclass correlation coefficient analysis for inter-rater reliability among five clinical experts.

Model	Accuracy	Completeness	Comprehensibility
ChatGPT 5	0.794	0.681	0.733
Claude Sonnet4.0	0.758	0.764	0.789
DeepSeek-R1	0.738	0.722	0.768
Gemini2.5 Pro	0.744	0.618	0.800
Grok-4	0.706	0.649	0.819

In the dimension of accuracy, ChatGPT (0.794) and Claude (0.758) showed good reliability, while Gemini (0.744), DeepSeek (0.738), and Grok (0.706) exhibited moderate reliability. In terms of completeness, Claude (0.764) displayed good reliability, while DeepSeek (0.722), ChatGPT (0.681), Grok (0.649), and Gemini (0.618) all fell within the moderate reliability range. In the comprehensibility dimension, DeepSeek (0.768), Gemini (0.800), Grok (0.819), and Claude (0.789) performed well, demonstrating good reliability, while ChatGPT (0.733) was classified as having moderate reliability.

Overall, the ICC results indicate that the evaluators were highly consistent in scoring each model across the three dimensions. This finding not only validates the robustness of the evaluation methodology used in this study but also provides a reliable measurement foundation for further in-depth analysis of the differences in the medical question-answering capabilities of the models.

### 3.4 Overall performance comparison of the five large language models

We conducted a systematic evaluation of the performance of the five LLMs in the field of medical question answering. The performance of each model across different dimensions is presented in Table 2.

**Table 2:** Overall performance comparison of five LLMs across the evaluation dimensions.

Model	Accuracy	Completeness	Comprehensibility
ChatGPT 5	4.36 ± 0.493	4.44 ± 0.510	4.25 ± 0.510
Claude Sonnet4.0	4.32 ± 0.485	4.27 ± 0.523	4.43 ± 0.532
DeepSeek-R1	4.23 ± 0.496	4.30 ± 0.538	4.37 ± 0.560
Gemini2.5 Pro	4.39 ± 0.500	4.10 ± 0.410	4.45 ± 0.498
Grok-4	4.22 ± 0.454	4.20 ± 0.442	4.30 ± 0.576

#### 3.4.1 Accuracy

In the accuracy assessment, the Friedman test results indicated a statistically significant difference among the five models (*P* = 0.015). The average ranks for each model were as follows: Gemini had the highest average rank of 3.45, followed by ChatGPT (3.12) and Claude (3.01), while DeepSeek and Grok had lower average ranks of 2.82 and 2.60, respectively. To further analyze these differences, we conducted pairwise comparisons, applying the Bonferroni correction. This revealed a significant difference between Grok and Gemini (*P* = 0.021), indicating that Gemini performed significantly better than Grok in terms of accuracy. Comparisons between the other models did not reach statistical significance.

#### 3.4.2 Completeness

In the completeness assessment, there were also significant differences in performance among the five models. The results of the Friedman test indicated (*P* < 0.001). The average ranks for each model were 3.78, 3.12, 3.20, 2.68, and 2.22 for ChatGPT, Claude, DeepSeek, Grok, and Gemini, respectively. The pairwise comparison results revealed a significant difference between Gemini and Claude (*P* = 0.011). In addition, Gemini displayed a significant disadvantage in comparison with DeepSeek (*P* = 0.004) and ChatGPT (*P* < 0.001). Furthermore, ChatGPT significantly outperformed Grok in terms of completeness (*P* = 0.001), further supporting its advantage in completeness performance.

#### 3.4.3 Comprehensibility

In the comprehensibility assessment, there were also significant differences among the models (*P* = 0.021). The average ranks indicated that both Claude and Gemini performed the best, each with an average rank of 3.31, while DeepSeek had an average rank of 2.98, Grok had an average rank of 2.82, and ChatGPT had the lowest average rank of 2.58. Despite the standout performance of Claude and Gemini in terms of comprehensibility, the subsequent pairwise comparison results did not reveal any statistically significant differences among the models.

#### 3.4.4 Overall assessment

**Figure 4:**
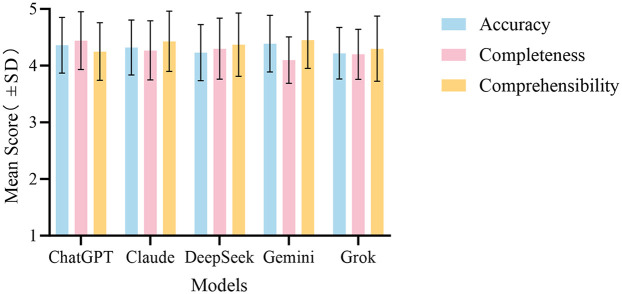
Overall performance comparison of the five LLMs in answering medical questions about esophageal cancer across the evaluation dimensions. The bar chart shows the mean scores ± (standard deviation) for ChatGPT, Claude, DeepSeek, Gemini, and Grok for accuracy, completeness, and comprehensibility.

In summary, the evaluation results reveal that Gemini excels in accuracy but has shortcomings in completeness. In contrast, ChatGPT demonstrates a more balanced overall performance, particularly excelling in the completeness assessment. The overall performance comparison of the five LLMs across the three evaluation dimensions is illustrated in Figure 4.

### 3.5 Performance analysis by medical question categories

We further evaluated the performance of the five LLMs in answering questions related to esophageal cancer, quantifying scores across four categories of medical questions, as shown in Table 3. We employed the Friedman test to compare the performances of the five models across the three dimensions in different question categories.

**Table 3:** Performance comparison of five LLMs across different question categories related to EC.

Problem Category	Evaluation dimension	ChatGPT	Claude	DeepSeek	Gemini	Grok
Basic knowledge of EC	Accuracy	4.34 ± 0.476	4.30 ± 0.537	4.45 ± 0.501	4.24 ± 0.484	4.25 ± 0.464
	Completeness	4.43 ± 0.497	4.29 ± 0.578	4.45 ± 0.501	4.17 ± 0.471	4.25 ± 0.436
	Comprehensibility	4.35 ± 0.530	4.40 ± 0.542	4.49 ± 0.528	4.34 ± 0.476	4.31 ± 0.518
Preoperative preparation	Accuracy	4.42 ± 0.499	4.20 ± 0.404	4.32 ± 0.471	4.65 ± 0.485	4.30 ± 0.463
	Completeness	4.28 ± 0.536	4.32 ± 0.471	4.52 ± 0.505	4.02 ± 0.319	4.12 ± 0.385
	Comprehensibility	4.10 ± 0.416	4.38 ± 0.490	4.52 ± 0.505	4.40 ± 0.495	4.34 ± 0.557
Surgery	Accuracy	4.46 ± 0.501	4.38 ± 0.488	4.11 ± 0.425	4.43 ± 0.498	4.18 ± 0.437
	Completeness	4.57 ± 0.498	3.31 ± 0.507	4.11 ± 0.535	4.13 ± 0.419	4.11 ± 0.449
	Comprehensibility	4.18 ± 0.525	4.39 ± 0.570	4.34 ± 0.612	4.52 ± 0.502	4.15 ± 0.668
Postoperative management	Accuracy	4.25 ± 0.479	4.33 ± 0.473	4.13 ± 0.506	4.34 ± 0.476	4.18 ± 0.458
	Completeness	4.42 ± 0.496	4.21 ± 0.518	4.24 ± 0.515	4.07 ± 0.383	4.28 ± 0.451
	Comprehensibility	4.30 ± 0.503	4.52 ± 0.502	4.23 ± 0.529	4.49 ± 0.502	4.40 ± 0.512

Data are presented as mean ± standard deviation.

#### 3.5.1 Accuracy in the question categories

In the accuracy assessment, the results showed that there were no statistically significant differences among the models in the categories of basic knowledge about esophageal cancer, preoperative preparation, and postoperative management (*P* = 0.218, 0.136, and 0.150, respectively), indicating that the accuracy performances of the five models in these areas were comparable.

However, a significant difference was observed in the surgery-related category (*P* = 0.033), with average ranks of 3.58, 3.18, 2.37, 3.42, and 2.45 for ChatGPT, Claude, DeepSeek, Gemini, and Grok, respectively. This indicates that ChatGPT performed best in this category, while DeepSeek performed relatively poorly. To further clarify the specific differences among the models in the surgery-related questions, we conducted pairwise comparisons using the Wilcoxon signed-rank test, which showed no significant differences among the models.

#### 3.5.2 Completeness in the question categories

In the completeness assessment, the five models did not show significant statistical differences in the category of basic knowledge about esophageal cancer (*P* = 0.068). However, significant statistical differences were observed in the preoperative preparation (*P* = 0.011), surgery-related questions (*P* < 0.001), and postoperative management (*P* = 0.007) categories.

In the preoperative preparation category, DeepSeek achieved the highest average rank of 4.10, followed by ChatGPT (3.35), Claude (3.20), and Grok (2.45), while Gemini had the lowest average rank (1.90). Pairwise comparisons indicated a significant difference between Gemini and DeepSeek (*P* = 0.019), showing that DeepSeek outperformed Gemini in completeness. No significant differences were found among the other models.

For the surgery-related questions, the average ranks were 4.11, 3.39, 2.58, 2.47, and 2.45 for ChatGPT, Claude, DeepSeek, Gemini, and Grok, respectively. ChatGPT excelled in this category, while Gemini and Grok performed relatively poorly. Further pairwise comparison results showed that ChatGPT significantly outperformed Grok (*P* = 0.012), Gemini (*P* = 0.015), and DeepSeek (*P* = 0.029), indicating ChatGPT's absolute advantage in the completeness dimension for this category.

In the postoperative management category, the Friedman test results indicated average ranks of 3.85, 2.95, 3.10, 2.08, and 3.03 for ChatGPT, Claude, DeepSeek, Gemini, and Grok, respectively. ChatGPT again exhibited the best performance, with Gemini ranking the lowest. The pairwise analysis revealed that ChatGPT had a significant advantage over Gemini (*P* = 0.004), while no significant differences were observed among the other models.

#### 3.5.3 Comprehensibility in the question categories

In the comprehensibility dimension, significant differences were not observed in three categories, with surgery-related questions the exception. The Friedman test results indicated no statistical significance for the basic knowledge of esophageal cancer (*P* = 0.803), preoperative preparation (*P* = 0.245), and postoperative management (*P* = 0.202) categories. However, there was a significant difference in comprehensibility for the surgery-related questions (*P* = 0.028) category. The average ranks showed that Gemini excelled in this category with an average rank of 3.79, followed by Claude (3.34), DeepSeek (2.95), ChatGPT (2.55), and Grok (2.37), with the latter having the worst performance. Nevertheless, the pairwise analysis revealed that, after applying the Bonferroni correction, none of the models reached the adjusted significance level.

## 4 Discussion

This study systematically evaluated the responses of five contemporary LLMs (ChatGPT-5, Claude Sonnet 4.0, DeepSeek-R1, Gemini 2.5 Pro, and Grok-4) to esophageal cancer questions using a three-dimensional rubric (accuracy, completeness, and comprehensibility). All the models achieved average scores >4.0/5 across these dimensions, indicating that current LLMs can reliably process oncological information and offer acceptable preliminary patient guidance, consistent with prior reports (22).

In the overall performance comparison, Gemini ranked highest for accuracy but underperformed in completeness relative to ChatGPT, Claude, and DeepSeek. This pattern likely reflects differing training emphases; for example, Gemini favors conservative, precision-oriented outputs, while models such as ChatGPT that benefit from RLHF tend to produce broader, more comprehensive responses (23). Although Grok performed well in benchmark tests, its lower overall ranking in this study may reflect limitations in its reasoning-oriented design when addressing specific oncology-related questions (24).

The performance analysis across different question categories indicates that the inter-model variance was mainly driven by completeness, while accuracy and comprehensibility were broadly comparable. For basic knowledge items (etiology and epidemiology), the models performed similarly (*P* > 0.05), suggesting that standardized medical knowledge has been widely integrated into the training data, providing a reliable foundation for patient education (25). However, significant differences in completeness were noted in the preoperative preparation, surgery-related questions, and postoperative management categories. This may be the result of the increased complexity of these questions, as the surgery and postoperative categories involve multidisciplinary integration and personalized recommendations, requiring enhanced reasoning and knowledge integration capabilities from the models.

The retesting results further reveal the dynamic performance of the models, with both Claude and DeepSeek showing significant improvements across all dimensions (*P* < 0.05). This may stem from the models' incremental learning mechanisms or parameter adjustments, indicating the potential of LLMs to enhance their performance through iterative optimization. Nevertheless, some questions still resulted in poor responses upon retesting, exposing potential issues within the models.

This study identified four notable questions that elicited poor responses, including two questions from the Claude model and one each from DeepSeek and Grok. The questions from Claude were “How common is esophageal cancer?” and “What can I expect on my body after esophagectomy?” Although the retest scores improved, the simplification of the content resulted in insufficient depth, hindering users' understanding of essential background knowledge and clinical significance. Models should maintain sufficient detail while being concise to fully address users' informational needs. In addition, incorporating regional epidemiological data is recommended to reduce geographic bias in the answers.

In the retest of the DeepSeek model, the question “How can treatment-related side effects be managed?” performed poorly in accuracy, primarily because the response did not align with the question's core focus. Patients seek specific, actionable recommendations for managing side effects; however, the provided information on preoperative and postoperative care was largely background-oriented and would not address patients' concerns directly. Furthermore, the descriptions of potential side effects lacked targeted management strategies, rendering the information vague and impractical.

After the retest for the question “Does an esophageal stent impact your radiation treatment plan for a patient with non-metastatic GE junction adenocarcinoma?” in Grok, the comprehensibility score decreased from 3.0 to 2.6. The retest responses featured many technical terms without adequate explanations, making comprehension difficult for healthcare professionals. In addition, the content lacked a clear logical structure, underemphasizing key information and reducing overall coherence. These issues align with the findings of Cross et al., highlighting the variability in the quality of AI-generated responses to medical inquiries (26).

Furthermore, data generated by LLMs has a significant risk of hallucinations, which refers to inaccurate, fabricated, or misleading information produced during content generation. This issue is particularly critical in the medical field. Research shows that approximately 30% of LLM responses to medical questions may exhibit some degree of hallucination (27, 28). To mitigate this risk, this study utilized expert review and multi-model validation. The medical experts assessed the generated content to identify any potential hallucinatory information and assign accuracy ratings. In addition, cross-validation among multiple LLMs helped reduce biases common to reliance on a single model. Future research should consider incorporating knowledge-based fact-checking tools to verify the accuracy of LLM-generated content, improving the identification of hallucinations. User education is also vital in promoting the safe use of LLM-generated information. Emphasizing that such content should be treated as a reference, not a substitute for professional medical advice, can increase awareness of potential risks. Implementing these measures will effectively address hallucination risks in LLM outputs, enhancing safety and reliability in medical applications and supporting the sustainable use of LLMs in clinical practice.

This study had several limitations. First, the subjectivity inherent in expert ratings must be acknowledged; although the ICCs indicated good reliability, cultural and experiential biases may affect consistency. Second, the question set was limited to 65 items and did not encompass all clinical variations, such as rare complications. Future studies should expand the sample size to enhance generalizability. Third, the rapid iteration of model versions means that current results may not apply to subsequent updates. In addition, the impact of multimodal inputs, such as imaging, was not assessed, limiting support for comprehensive clinical decision-making. Finally, this study did not involve real patient interactions; future research should include patient satisfaction surveys to validate the practical utility of the findings.

In summary, this study provides an important empirical foundation for clinical applications of AI in medicine, revealing the strengths and weaknesses of different models and their suitable contexts. As AI technology continues to evolve, we have reason to believe that through thoughtful selection and application strategies, large language models will increasingly play a vital role in enhancing patients’ healthcare experiences and improving the quality of medical services.

## Data Availability

The raw data supporting the conclusions of this article will be made available by the authors, without undue reservation.

## Supplementary material

Supplementary Table S1 Sources and examples of common patient questions on esophageal cancer, including the official names of websites or journals, corresponding URLs, and the specific questions collected.

Supplementary Table S2 Initial responses from five LLMs to 65 patient questions, listing each question alongside the corresponding model-generated answers.

Supplementary Table S3 Retest questions and corresponding answers for each of the five LLMs.
